# ROR1 is upregulated in endometrial cancer and represents a novel therapeutic target

**DOI:** 10.1038/s41598-020-70924-z

**Published:** 2020-08-17

**Authors:** Dongli Liu, Kate Gunther, Luis A. Enriquez, Benjamin Daniels, Tracy A. O’Mara, Katrina Tang, Amanda B. Spurdle, Caroline E. Ford

**Affiliations:** 1 Gynaecological Cancer Research Group, Lowy Cancer Research Centre, School of Women’s and Children’s Health, Faculty of Medicine, University of New South Wales, Sydney, NSW 2052 Australia; 2 Medicines Policy Research Unit, Centre for Big Data Research in Health, Faculty of Medicine, University of New South Wales, Sydney, Australia; 3 QIMR Berghofer Medical Research Institute, Brisbane, Australia; 4 South Eastern Area Laboratory Services Pathology, Prince of Wales Hospital, Sydney, Australia

**Keywords:** Cancer, Cell biology, Oncology

## Abstract

ROR1 and ROR2 are receptor tyrosine kinases with altered expression in a range of cancers. Silencing ROR1 or ROR2 in different tumour types has been shown to inhibit proliferation and decrease metastatic potential. The aim of this study was to investigate the role of ROR1 and ROR2 in endometrial cancer via immunohistochemistry (IHC) in a large endometrial cancer patient cohort (n = 499) and through in vitro analysis in endometrial cancer cell lines. Correlation was assessed between ROR1/2 expression and clinicopathological parameters. Kaplan Meier curves were produced for 5-year progression free survival (PFS) and overall survival (OS) with low/moderate versus high ROR1/2 intensity. Cox multivariate regression was applied to analyse the effect of selected covariates on the PFS and OS. The effect of ROR1 and/or ROR2 modulation on cell proliferation, adhesion, migration and invasion was analysed in two endometrial cancer cell lines (KLE and MFE-296). We observed a significant decrease in OS and PFS in patients with high ROR1 expression. ROR1 silencing and ROR2 overexpression significantly inhibited proliferation of KLE endometrial cancer cells and decreased migration. This study supports the oncogenic role of ROR1 in endometrial cancer, and warrants investigation of future application of ROR1-targeting therapies in endometrial cancer patients.

## Introduction

Endometrial cancer (EC) is the most prevalent gynaecological cancer and the sixth most common malignancy worldwide^1^. Incidence has increased significantly over the last decade, particularly in developed countries^2^. This escalating worldwide burden and poor survival outcomes from advanced stage and aggressive subtypes warrants further research into novel targets and new therapies.

The pathogenesis for EC is multifactorial, with risk factors including genetic variants^3^, high BMI^4,5^, high number of cumulative menstrual cycles^6,7^, and infertility^8^. In 1983, Bokhman^9^ proposed the classic dualistic model which divided EC into estrogen driven endometrioid subtype (Type I) and the more aggressive non-endometrioid subtype (Type II). Based on the histopathological features, EC is also commonly classified into endometrioid adenocarcinoma, serous carcinoma, mucinous carcinoma, clear cell carcinoma mixed carcinoma etc.^10^. There are certain overlaps between the two classification systems: Type I is generally endometrioid subtype and Type II is mostly serous. These traditional classification systems based on endocrine or histopathological features failed to take into account the heterogeneity of EC and were limited due to technical difficulties and controversies in histopathological assessment^11,12^. In 2013, the Cancer Genome Atlas (TCGA) defined four genomic subgroups: Polymerase epsilon (*POLE)*-mutant tumours (ultrahypermutated), MSI (hypermutated), copy-number low (endometrioid) and copy-number high tumours (serous-like) through integration of multi-omics data^13^. Although this system is not yet in widespread clinical use, the identification of molecular targets correlate to disease progression and development of treatment could hold translational importance.

The Wnt signalling pathway is generally divided into two arms—the canonical pathway (β-catenin dependent) and non-canonical pathway (β-catenin independent), which both have been implicated in a range of human cancers^14^. β-catenin somatic mutations are common in the endometrioid subtype of EC^15–17^ but this pathway has not yet been successfully targeted therapeutically in EC. One potential avenue to target Wnt signalling may be via the recently identified Wnt receptors, ROR1 and ROR2.

ROR1 and ROR2 are tyrosine kinase-like orphan receptors that play critical roles in embryogenesis. Aberrant expression of ROR1 has been observed in a range of cancers^18–23^ compared to its limited expression in healthy adult tissue, which made it a candidate target for treating these cancers. ROR1 has been demonstrated to play an oncogenic role in many tumour types and has been broadly linked with cell proliferation, stemness^24^, the epithelial-mesenchymal transition (EMT)^25^ and other metastatic abilities^26^. In contrast, the role of ROR2 in carcinogenesis remains controversial as it acts as either a tumour suppressor or tumour promoter in different cancers^27,28^. ROR2 can also function as an inhibitor of the canonical Wnt pathway^29^. The interaction between the two receptors in Wnt signalling remains unclear. Wnt5a has been shown to induce the ROR1/ROR2 heterooligomers to activate signalling in chronic lymphocytic leukaemia (CLL), and neither ROR1 nor ROR2 alone was efficient in triggering the optimal downstream cascade^30^. Currently it is unclear if this heterodimer is formed for all cancer types.

In ovarian cancer, we have demonstrated that both ROR1 and ROR2 are overexpressed in large cohorts of tumour tissue^26^, and that silencing ROR1 and ROR2 inhibits metastatic potential^26^, which supported the oncogenic role of the two receptors. In contrast, when we conducted a similar study in EC of limited sample size (n = 87), we identified potential distinct roles for ROR1 and ROR2^31^. The aim of this study was to investigate the role of ROR1 and ROR2 in EC in a larger Australian population-based EC cohort, encompassing all major subtypes of the disease, and to perform a series of *in-vitro* experiments to clarify the role of each receptor.

## Results

**Figure 1:**
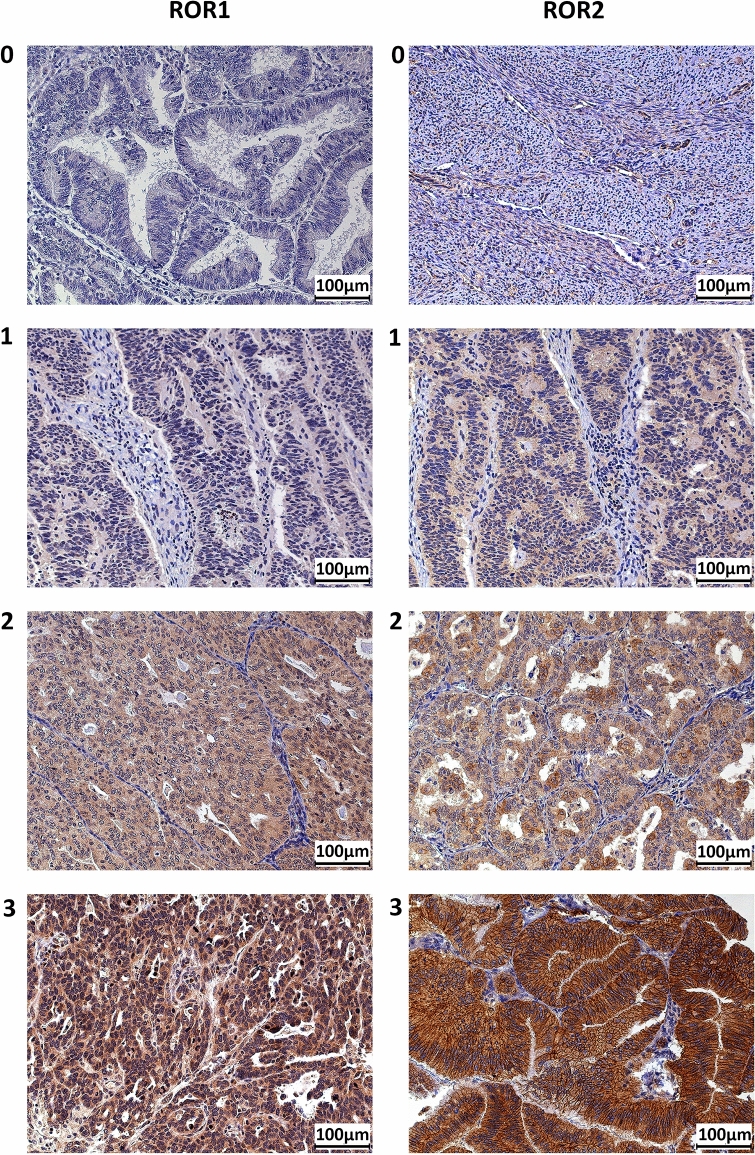
ROR1 and ROR2 protein expression as measured by immunohistochemistry. Representative images of score 0 (absence), 1 (weak), 2 (moderate), 3 (intense) for both ROR1 and ROR2.

Overall the clinical cohort showed a broad range of expression levels for both ROR1 and ROR2 (Fig. 1, Supplementary Fig. S1). Compared to the tumour tissue, normal samples showed lower expression of ROR1 or ROR2 (Supplementary Fig. S1). None of the normal tissue was scored as high (i.e. 3) for either ROR1 or ROR2. Over 90% of the normal tissue had ROR1 or ROR2 stained less than 2 (Supplementary Fig. S1A,B). For the matched normal and tumour tissues (n = 19), the expression level of ROR1 or ROR2 was significantly different between tumour and adjacent normal tissues (Supplementary Fig. S1C,D).

### ROR1 correlates with clinicopathological parameters

**Figure 2:**
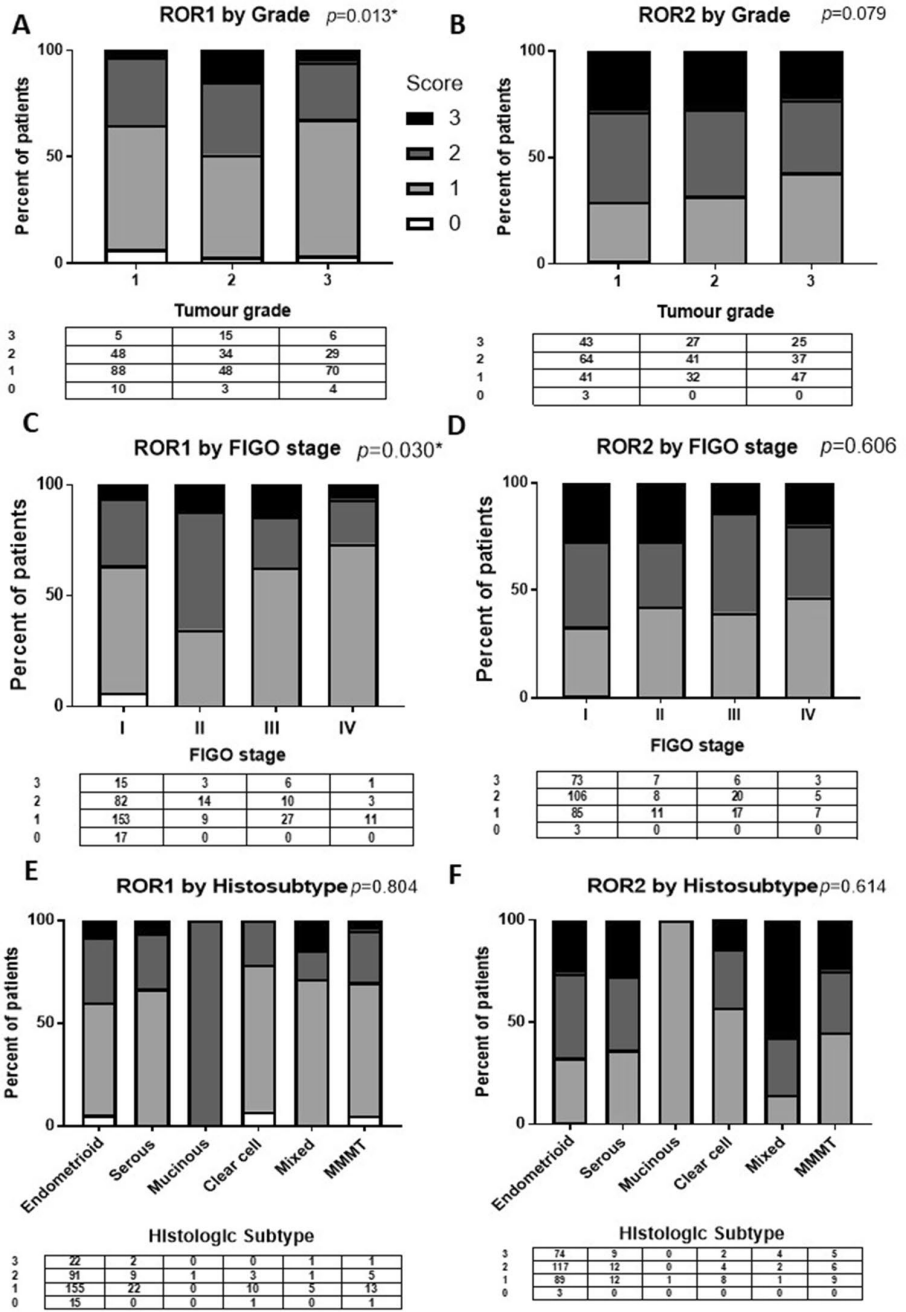
ROR1 expression was significantly correlated with tumour grade and International Federation of Gynecology and Obstetrics (FIGO) stage in endometrial cancer. (**A**) Expression of ROR1 in endometrial cancer stratified by tumour grade. The values in the table below showed the number of score 0, 1, 2, 3 in each grade. *P* values resulted from Chi-square or Fisher’s exact test indicated the significant level of the correlation. (**B**) Expression of ROR2 in endometrial cancer stratified by tumour grade. (**C**) Expression of ROR1 in endometrial cancer stratified by FIGO stage. (**D)** Expression of ROR2 in endometrial cancer stratified by FIGO stage. (**E**) Expression of ROR1 in endometrial cancer histologic subtypes including endometrioid, serous, mucinous, clear cell, mixed and malignant mixed mesodermal tumour (MMMT); expressed as a percentage of total. F: Expression of ROR2 in endometrial cancer subtypes. *Significant at *p* < 0.05.

Among the clinical cohort (n = 360), ROR1 expression level was significantly associated with tumour grade (*p* = 0.013) and International Federation of Gynecology and Obstetrics (FIGO) stage (*p* = 0.030) (Fig. 2A,C). No significance was observed between ROR1 expression and histologic subtype (Fig. 2E) or ROR2 with any of the three parameters (Fig. 2B,D,F).

In the endometrioid EC patients, the expression level of ROR1 was significantly correlated with tumour grade (*p* = 0.019, Supplementary Fig. S2).

### ROR1 correlates with shorter OS and PFS

**Figure 3:**
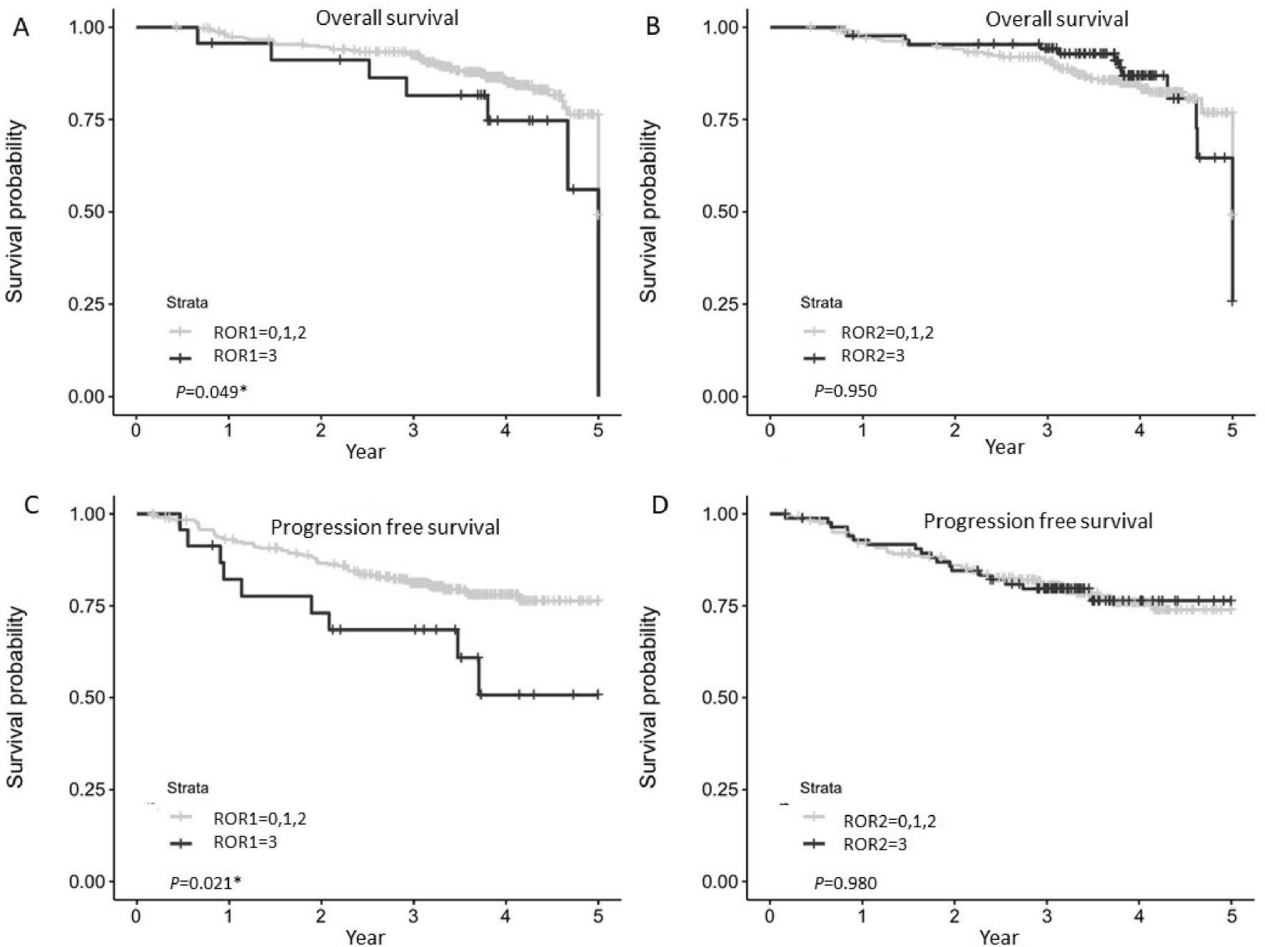
Kaplan–Meier analysis for ROR1 and ROR2 stratified by low/moderate (score 0, 1, 2) and high (score 3) in the complete cohort (n = 330). (**A**) Overall survival (OS) according to ROR1 expression. (**B**) Progression free survival (PFS) according to ROR1 expression. (**C**) OS according to ROR2 expression. (**D**) PFS according to ROR2 expression. *Significant at *p* < 0.05.

A significant decrease in endometrial cancer specific OS and PFS was observed in patients with high ROR1 expression (*p* = 0.049 and *p* = 0.021, respectively, in Fig. 3) in the clinical cohort. No significant correlation was observed for ROR2 expression on OS or PFS, however patients with high ROR2 showed a trend towards better PFS.

Compared to the low ROR1 expressed patients, moderate and high ROR1 was not significantly correlated with OS or PFS (Supplementary Fig. S3A,C). No significant correlation was observed for low ROR2 expression with OS or PFS (Supplementary Fig. S3B,D).

In terms of the multivariate parameters associated with OS and PFS for the analytical cohort (Table 1), the FIGO stage and tumour grade was significantly associated with both OS and PFS significantly. ROR1 level was significantly associated with OS and PFS while ROR2 was not significant. Compared to the low or moderate level of ROR1 expression, high ROR1 had a significantly increased risk of EC related death and relapse (hazard ratio = 2.48 and 2.45 respectively).

**Table 1:** Multivariate analyses of parameters associated with overall survival (OS) and progression free survival (PFS).

Parameter	Value	HR^a^	HR lower 95CI	HR upper 95CI	*P* value
**OS**					
Age	≤ 50 versus > 50	0.34	0.04	2.68	0.31
BMI	> 30 versus ≤ 30	1.37	0.72	2.59	0.33
FIGO stage	II versus I	0.55	0.13	2.37	0.42
FIGO stage	III, IV versus I	3.71	1.91	7.21	< .0001*
Tumour grade	2 versus 1	4.14	1.13	15.14	0.03*
Tumour grade	3 versus 1	17.5	4.91	62.43	< .0001*
Subtype	Nonendo versus Endo	0.65	0.3	1.45	0.30
ROR1	High versus low/moderate	2.48	0.99	6.18	0.05*
ROR2	High versus low/moderate	0.77	0.34	1.72	0.52
**PFS**					
Age	≤ 50 versus > 50	0.42	0.1	1.77	0.24
BMI	> 30 versus ≤ 30	0.80	0.48	1.33	0.39
FIGO stage	II versus I	0.91	0.35	2.35	0.85
FIGO stage	III, IV versus I	4.25	2.43	7.43	< .0001*
Tumour Grade	2 versus 1	1.50	0.75	3.02	0.25
Tumour Grade	3 versus 1	5.81	2.83	11.92	< .0001*
ROR1	High versus low/moderate	2.45	1.21	4.97	0.01*
ROR2	High versus low/moderate	0.92	0.51	1.67	0.78

^a^Hazard ratio.

*Significant at *p* < 0.05 level.

### ROR1 silencing and ROR2 overexpression inhibit tumour progression in KLE EC cells

**Figure 4:**
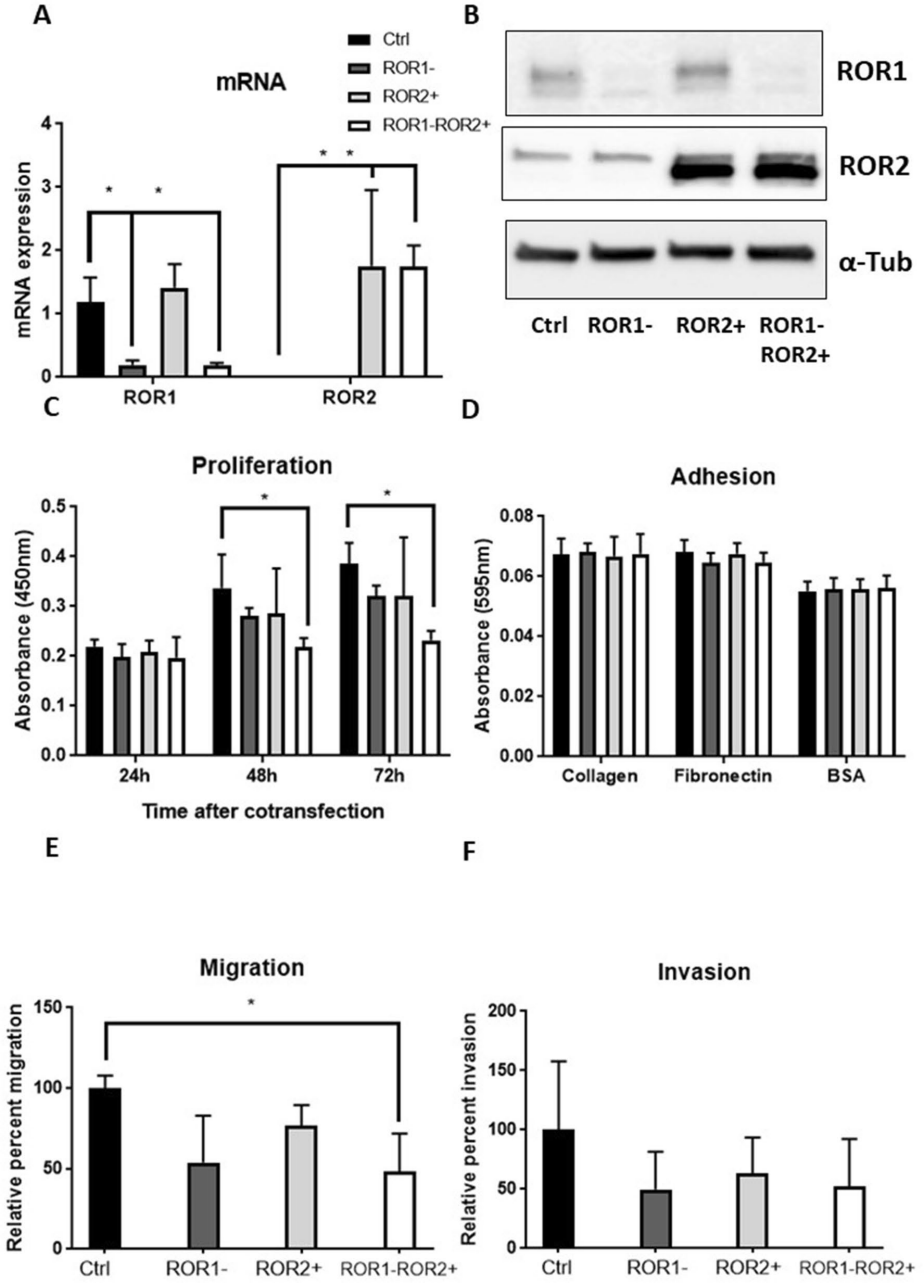
ROR1 knockdown and ROR2 overexpression significantly decreased proliferation and migration of KLE. (**A**) ROR1 mRNA expression level was reduced significantly without changing ROR2 following single ROR1 siRNA transfection. ROR2 mRNA expression level was elevated significantly with no changes in ROR1 mRNA level following single ROR2 plasmid transfection. Cotransfecting ROR1 siRNA and ROR2 plasmid significantly reduced ROR1 while increased ROR2 at mRNA level. (**B**) Representative western blot membranes showed effective delivery of ROR1 siRNA and/or ROR2 plasmid in KLE. (**C**) ROR1 knockdown and ROR2 overexpression significantly reduced the cell proliferation after 48 h and 72 h (*p* = 0.043 and 0.004 respectively). (**D**): ROR1 knockdown and/or ROR2 overexpression had no effect on adhesion to collagen or fibronectin. (**E**): ROR1 knockdown and ROR2 overexpression decreased KLE migration ability significantly (*p* = 0.037). (**F**) No significant change was observed for invasion following ROR1 knockdown and/or ROR2 overexpression. For all panels n = 3, error bars represent standard deviation of the mean, *p < 0.05.

The high ROR1, low ROR2 expressing KLE cell line was chosen as a model for serous EC. After 48 h, the transfection was shown to be effective at both transcription and translation levels (Fig. 4A,B). ROR1 knockdown decreased proliferation after 72 h but was not statistically significant (*p* = 0.071). The combination of ROR1 knockdown and ROR2 overexpression further reduced the cell proliferation significantly after 48 h and 72 h (Fig. 4C, *p* = 0.043 and 0.004 respectively). ROR2 overexpression reduced migration moderately (*p* = 0.059), and this reduction was enhanced (Fig. 4E, *p* = 0.037) when combining with ROR1 knockdown. No significant change was observed in adhesion or invasion assays (Fig. 4D,F).

### ROR2 silencing and ROR1 overexpression play distinct roles in MFE-296 EC cells

**Figure 5:**
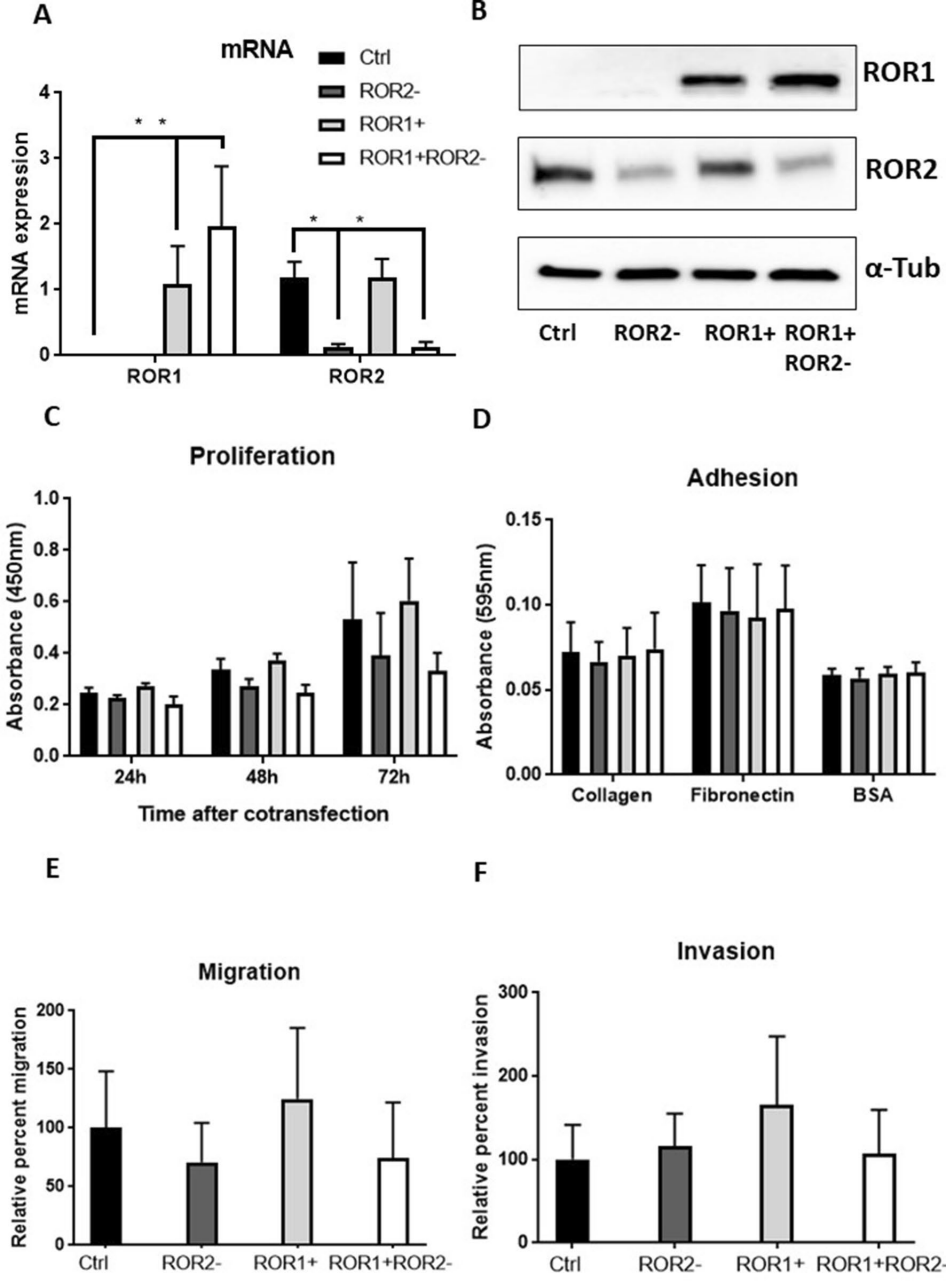
ROR1 overexpression and ROR2 knockdown play different roles in MFE-296. (**A**) ROR2 mRNA level was reduced significantly without changing ROR1 following single ROR2 siRNA transfection. ROR1 mRNA level was increased significantly with no change in ROR2 following single ROR1 plasmid transfection. Cotransfecting ROR2 siRNA and ROR1 plasmid significantly reduced ROR2 while increased ROR1 at mRNA level. (**B**) Representative western blot membranes showed effective delivery of ROR2 siRNA and/or ROR1 plasmid in MFE-296. (**C**) No significant change of proliferation was observed after 48 h or 72 h following ROR1 overexpression and/or ROR2 knockdown. (**D**) ROR2 knockdown and/or ROR1 overexpression had no effect on adhesion to collagen or fibronectin. (**E**) ROR1 knockdown and/or ROR2 overexpression did not change MFE-296 cell migration significantly. (**F**) No significant change was observed for invasion following ROR2 knockdown and/or ROR1 overexpression. For all panels n = 3, error bars represent standard deviation of the mean, *Significant at *p* < 0.05.

The high ROR2, low ROR1 expressing MFE-296 cell line was chosen as a model for endometrioid EC. The results from qRTPCR and Western blot indicated ROR2 was suppressed after ROR2 siRNA transfection, ROR1 was elevated following ROR1 plasmids transfection (Fig. 5A,B). ROR1 overexpression or ROR2 silencing showed opposite effects on cell proliferation and migration (Fig. 5C,E). ROR1 overexpression seemed to increase cell proliferation while ROR2 knockdown tended to decrease cell proliferation. The combination of the two showed average lower proliferation ability compared to the control. Similarly, ROR1 overexpression tended to increase cell migration while ROR2 knockdown showed an opposite trend. ROR1 overexpression showed a higher average invaded cell number compared to control (Fig. 5F). However, none of these observations were significant at 0.05 level. No significant change was observed in adhesion after ROR1 overexpression or/and ROR2 knockdown (Fig. 5D).

## Discussion

This study confirms ROR1 as a potential therapeutic target in EC. Despite the different ROR1 primary antibody used in the IHC compared to our previous smaller cohort^31^, we have shown the same effect of ROR1 on OS and PFS in our clinical cohort. Patients with high ROR1 expression have a significant lower OS and PFS compared to those with low or moderate level of ROR1 expression. The new anti-ROR1 antibody we have used in this study is a monoclonal antibody validated in various clinical cohorts^18^, as opposed to the polyclonal antibody used in our previous publications^31^. We identified the same association of ROR1 with higher grade in EC, as reported previously in ovarian cancer and pancreatic cancer^18^. The effect of ROR1 on OS or PFS was not significant when stratified as low and moderate/high.

In contrast, ROR2 appears to play a less important role in EC. Previously we reported a moderately negative correlation with OS (*p* = 0.06) in a small patient cohort of 87 EC patients. However, this trend was not observed when we expanded the sample size to 341. ROR2 seems to play a less important role in terms of survival or progression. However, we found a moderate correlation between ROR2 expression and tumour grade in our cohort (*p* = 0.079, Fig. 2B). The Clinical Proteomic Tumor Analysis Consortium (CPTAC) Confirmation/Discovery cohort (n = 131)^32^ showed a significant reduction in expression of ROR2 as the tumour grade increased in EC (Supplementary Fig. S4). ROR2 can trigger the non-canonical pathway upon binding with Wnt5a^33^, or the canonical pathway by binding with Wnt3a^34,35^. ROR2 can also inhibit canonical Wnt signalling through interacting with Wnt5a^29^. Previous studies have reported an oncogenic role for ROR2 in osteosarcoma^36^, renal cell carcinoma^37^, and breast cancer^38^ while it presented a tumour suppressor role in colon cancer^39^ and hepatocellular carcinoma^40^. It was hypothesised that the role ROR2 played depend on which arm of Wnt signalling played dominant role in the cancer or specific subtype^41^. In cancers where canonical Wnt signalling is a key driver of the disease (eg through mutations in β-catenin or APC), such as colon cancer, ROR2 may play a major role in inhibiting the canonical pathway through binding to Wnt5a. In contrast, ROR2 may play a more direct role in triggering the noncanonical Wnt signalling pathway in noncanonical signalling driven cancers. EC is a complex case that requires further investigation into the overlapping roles of the two key Wnt pathways. While it is clear that canonical Wnt signalling is a key driver in the endometrioid subtype of ovarian cancer (due to the high % of β-catenin mutations), its role in other subtypes needs to be clarified.

ROR1 and ROR2 share the same ligand–Wnt5a^35,42^, and play essential roles in Wnt signalling associated metastasis. However, the relationship between the two receptors is not well established. Simultaneous knock-down of the two receptors showed a stronger effect than silencing either individually on reducing cell metastasis potential in ovarian cancer^26,43^. Unlike the ovarian cancer cell lines, most of the EC cell lines appear to express either ROR1 or ROR2 (Supplementary Fig. 2 from^31^). A previous study reported that ROR1 overexpression in MEC1 (a CLL cell line with high ROR2 no ROR1) induced the formation of ROR1/2 heterooligomers in the context of Wnt5a and enhanced subsequent non-canonical Wnt signalling cascade^30^. However, we observed no significant change in ROR2 expression in MFE-296 after increasing ROR1 levels in this study (Fig. 5A). In fact, no change of ROR1/2 was observed after modulating the other receptor in KLE or MFE-296. It could be hypothesised that no heterooligomer was formed for non-canonical Wnt signalling.

Single ROR1 silencing and ROR2 overexpression in KLE showed a similar trend in altering cell proliferation and migration. The combination of the two treatments further strengthened the effect. In low-ROR1 expressing MFE-296, ROR1 overexpression tended to increase proliferation and migration. But ROR2 silencing did not show the same trend, therefore neutralised the effect of ROR1 overexpression in the combination treatment. The epithelial-mesenchymal-transition (EMT) through which epithelial cells gain migratory and invasive properties and become mesenchymal status, serves as a critical step in regulating tumour metastasis^44,45^. Previous studies in ovarian cancer found ROR1 played a role in the EMT procedure^25,46^. Thus, it could be hypothesised that ROR1 also regulated the EMT in endometrial cancer and modulation in ROR1 could alter cell migration and invasion ability. In general, ROR1 promoted tumour growth and progression in EC cell lines in vitro. The role of ROR2 seemed to be different between endometrioid and non-endometrioid subtypes. It will be important to conduct further research using diverse EC cell lines derived from various subtypes to uncover the role of ROR2. In addition, general extracellular matrix component precoated plates or transwell membranes could not represent the real inner environment for the tumour cells to attach or invade through. Further research into 3D culture or animal models is needed to validate the influence of ROR1/2 modulation on cell adhesion and invasion.

Not only was ROR1 functionally relevant to EC tumorigenesis and progression, ROR1 expression was found to be significantly increased in EC tumour tissue compared to normal tissue (Supplementary Fig. S1). Combined with the survival data this suggests that ROR1 is a promising therapeutic target in EC. There are a number of ROR1 targeting therapies currently in development or in early phase trials. Cirmtuzumab is a monoclonal antibody that targets and inhibits ROR1. It was developed by the Kipps lab, UCSD, originally focused on Chronic Lymphocytic Leukemia (CLL). It has proven to be effective in inhibiting ROR1 signalling in preclinical trials for ovarian cancer^21^ and shown safe in a phase I trial to treat CLL^47^. It is currently being tested in phase Ib trial in triple negative breast cancer (NCT02776917). Another ROR1-targeting therapy which has been tested in clinical trials is the immunotherapy called ROR1 chimeric antigen receptor (CAR)-T cell therapy^48^. A phase I trial (NCT02706392) is currently recruiting ROR1 positive cancers such as CLL and triple negative breast carcinoma. These new treatments may also benefit EC patients, especially those with high ROR1 expression.

The synergistic effect noted here in vitro of ROR1 inhibition and ROR2 overexpression may suggest a more effective combination treatment for EC patients. However, there is no current treatment that specifically targets and promotes ROR2 expression. Recently, ROR2 was found to be epigenetically inactivated in colorectal cancer and demethylation treatment with 5-aza-2-deoxycytidine could restore the expression level of ROR2 in vitro^49^. The combination of ROR1 inhibition and demethylation might make the intervention more effective to some specific subgroups of EC patients.

This study confirms the role of ROR1 plays in endometrial cancer and warrants the future application of ROR1-targeting therapies in endometrial cancer patients. With endometrial cancer rates increasing rapidly worldwide there is a clear need for more treatment options for this patient group.

## Methods

All experimental protocols were approved by University of New South Wales (UNSW), Australia. Ethics approval was obtained from the UNSW Human Research Ethics Advisory Panel (#HC15771). All methods were carried out in accordance with relevant guidelines and regulations. Informed consent was obtained from all patients for the clinical cohort.

### Clinical cohort

The Australian National Endometrial Cancer Study (ANECS) is an Australia-wide population-based study that recruited women with histologically confirmed EC between 2005 and 2007^50^. Tumour tissue microarray (TMA) slides from the ANECS cohort were obtained from the QIMR Berghofer Medical Research Institute. The TMA cores included 578 cancer tissues, 36 adjacent normal tissues and 32 recurrent tumours from 499 individual patients. Among the 499 patients, 93 were excluded from analysis due to missing or insufficient tissue (< 40%), 39 were excluded for no epithelium observed in the TMA core, 7 were excluded for missing all clinicopathological data, which resulted in a clinical cohort of 360 individual cases for this study. The accompanying clinicopathological data including age (grouped into ≤ 50 and > 50), body mass index (BMI, grouped into ≤ 30 kg/m^2^ and > 30 kg/m^2^), FIGO stage (2009), histological subtype, tumour grade, menopause status, recurrence status and vital status etc. were provided by the ANECS and are summarised in Table 2.

**Table 2:** Demographic and clinicopathological characteristics of the tumour samples in the clinical cohort.

	Number of cases	Percentage (%)
**Age at diagnosis (y)**		
≤ 50	23	6.7
> 50	318	93.3
N/A^a^	19	-
**BMI (kg/m**^**2**^**)**		
≤ 30	179	53.0
> 30	159	47.0
N/A	22	–
**Tumour grade**		
1	151	41.9
2	100	27.8
3	109	30.3
**FIGO stage (2009)**		
I	267	76.1
II	26	7.4
III	43	12.3
IV	15	4.3
N/A	9	–
**Histological subtype**		
Endometrioid	283	79.1
Serous	33	9.2
Clear cell	14	3.9
Mucinous	1	0.3
MMMT^b^	20	5.6
Mixed	7	2.0
N/A	2	–
**Menopause status**		
Peri	14	4.1
Pre	32	9.4
Post	295	86.5
N/A	19	–

^a^Data not available, ^b^Malignant mixed Müllerian tumour.

### Immunohistochemistry

Immunohistochemistry (IHC) for ROR1 (1:50, #564464, BD Biosciences, USA) and ROR2 (1:100, #34045, QED Bioscience, USA) were performed using the Leica Bond RX system (Leica Microsystems, USA) at the Garvan Institute of Medical Research, Sydney Australia.

The intensity of ROR1/2 staining was graded as 0 (absence), 1 (weak), 2 (moderate) and 3 (intense) as previously described^31^. Representative images are shown in Fig. 1. The TMAs were scored independently and blinded by three researchers (DL, KG and LE) from the UNSW Gynaecological Cancer Research Group (GCRG) and a pathologist from the Prince of Wales Hospital (KT). The concordant scores were achieved by discussion with a fourth author (CF).

### Statistical analysis of the clinical cohort

Paired t-test (2-tails) was used to evaluate the difference of ROR1/2 expression between the matched normal and tumour tissue. Chi-square or Fisher’s exact test was used to analyse the association between ROR1 and ROR2 staining intensity and clinicopathological parameters including FIGO stage, grade, and subtypes. Spearman rank correlation coefficients (Spearman's rho) were calculated to show the direction of the relationship between two measures.

There were 30 patients from the clinical cohort (n = 360) who had specified non-EC related death or missing time-to-event data, which resulted in 330 cases (complete cohort) in the following survival analysis. The filtering process of the sample size is shown in Supplementary Fig. S5.

Kaplan Meier curves were produced for 5-year progression free survival (PFS) and overall survival (OS) with ROR1/2 intensity. The expression level of ROR1 and ROR2 were aggregated to low/moderate (score 0, 1, 2) and high (score 3), or low (score 0, 1) and moderate/high (score 2, 3). PFS was defined as the time (days) from diagnosis to recurrence or death. OS was defined from the diagnostic date to death.

Cox multivariate regression was also applied to analyse the impact of selected covariates (age, BMI, FIGO stage, tumour grade and histologic subtypes) on the PFS and OS. FIGO stage III and IV were aggregated together in the analysis. All subtypes were grouped into endometrioid and non-endometrioid groups. The log-rank test was used to evaluate the association between the covariates and PFS or OS.

All the analyses were performed by a trained biostatistician (BD) using SAS software, Version 9.4 of the SAS System for Unix. Copyright © 2016 SAS Institute Inc. Figures were provided in R (v3.6)^51^.

### Cell culture

EC cell lines MFE-296 (endometrioid) and KLE (serous) were a gift from Dr Frances Byrne and Associate Professor Kyle Hoehn (UNSW, Australia). KLE was maintained in DMEM/F12 medium and MFE-296 was cultured in MEM medium, both containing 10% foetal bovine serum (FBS), 1% GlutaMAX and 1% penicillin/streptomycin. Cells were grown in 5% CO_2_ at 37 °C and underwent mycoplasma testing once a month. All cells were shown to be free of contamination and were confirmed via cell line identification service at the Garvan Institute.

### Transfection treatment

For both KLE and MFE-296, four types of co-transfection were conducted using Lipofectamine2000 (Invitrogen, USA) according to the manufacturer’s protocol. For KLE (high ROR1, low ROR2), ROR1 silencing, ROR2 overexpression, ROR1 silencing in conjunction with ROR2 overexpression and negative control were performed. In contrast, ROR2 silencing, ROR1 overexpression, ROR2 silencing in conjunction with ROR1 overexpression and negative control were prepared for MFE-296 (high ROR2, low ROR1). We plated 5 × 10^5^ KLE or MFE-296 cells on 6-well plates and serum starved overnight before each treatment. ROR1 or ROR2 silencing was achieved via co-transfection with 90 pmol ROR1 siRNA (#s9755, Ambion, USA) or ROR2 siRNA (#s9759, Ambion, USA) as well as empty plasmid. ROR1 pCMV3 plasmid (#HG13968-NH, Sino Biological, China) or ROR2 pFLAG plasmid (previously used in^49^) and non-targeting siRNA (#4390844, Ambion, USA) were co-transfected for ROR1 or ROR2 overexpression. All the aforementioned conditions were compared to the negative control which was prepared by transfecting both non-targeting siRNA and empty plasmid.

### qRT-PCR

Total RNA was extracted and real-time RTPCR was performed as previously described^31^. The expression level of *ROR1*, *ROR2* was analysed. For each gene, non-reverse transcribed RNA samples were included as a negative control. The relative expression level of each gene was calculated using 2^–∆∆**Ct**^ method and normalised against the mean of three house-keeping genes (*HSPCB*, *SDHA*, *RPL13A*)^52^. Primer sequences were provided in^26^.

### Western blot

Total protein was extracted from the cells using cell lysis buffer (Cell Signalling Technology, USA) with protease inhibitor (Sigma-Aldrich, USA). 20 µg protein samples were separated on 4–20% Mini-PROTEAN TGX precast gels (Bio-rad, Australia) and transferred onto nitrocellulose membranes. 3% non-fat milk (Coles, Australia) in 0.1% Tween in Tris buffered saline (TBST) was used as blocking buffer and antibody diluent. The membranes were blocked for 1 h at room temperature before the overnight incubation with primary antibody at 4 °C. The primary antibodies used were monoclonal rabbit anti-ROR1 (#AF2000, R&D Systems, USA), monoclonal mouse anti-ROR2 (#34045, QED Bioscience, USA) and monoclonal mouse anti-α-Tubulin (#3873, Cell Signalling, USA). After washing with TBST, the membranes were incubated with either polyclonal rabbit anti-mouse immunoglobulins/HRP (#P0260, Dako, Denmark) or polyclonal rabbit anti-goat immunoglobulins/HRP (#P0449, Dako, Denmark) at 1:5,000 dilution for 1 h at room temperature. After another set of washes, the membranes were incubated with enhanced chemiluminescence (ECL) reagent and imaged on the ImageQuant LAS4000 system (GE Healthcare Life Sciences, USA). Full-length blots with multiple exposures were provided for ROR1 in Supplementary Fig. S6. Replicate blots for ROR2 were also provided instead of full-length as the blots were cropped to perform reference (α-Tubulin).

### Proliferation assay

Six hours following the transfection, the cells were plated in a 96-well plate at 4,000 cells per well and analysed with the Cell Counting Kit-8 (CCK-8, Sigma-Aldrich, USA) as per manufacturer protocol at 24 h, 48 h and 72 h after transfection.

### Adhesion assay

The adhesion assay was performed as previously described^31^. Briefly, cells adhering to 10 μg/ml type I collagen (Sigma-Aldrich, USA), 5 μg/ml fibronectin (Millipore, USA) or 3% bovine serum albumin (BSA) in PBS after 2 h were stained with 0.1% Crystal violet (Sigma-Aldrich, USA) and lysed with 50% acetic acid. The amount of cells attached was assessed using absorbance at 595 nm.

### Migration assay

The migration analysis was performed using the Corning transwell insert system according to manufacturer’s protocol (Corning Life Sciences, USA). Six hours after the transfection, the cells were trypsinized and plated in the inserts in triplicates (5 × 10^4^ cells per insert for KLE or MFE-296). After 48 h incubation, the migrated cells attached to the membranes were fixed with methanol, stained with 1% Crystal violet and imaged as previously described^31^.

### Invasion assay

Corning Matrigel pre-coated transwell inserts were used for invasion assays as per manufacturer’s protocol (Corning Life Sciences, USA). Six hours after the transfection, KLE and MFE-296 (1 × 10^5^ cells) were seeded in the inserts. The subsequent steps were the same as the migration assay.

### Statistical analysis of cell assays

All assays were repeated three times. The results were shown as mean ± standard deviation. Significance cut-off was set at *p* = 0.050.

## Supplementary information

Supplementary Figures.
