# Author Correction: ROR1 is upregulated in endometrial cancer and represents a novel therapeutic target

**DOI:** 10.1038/s41598-022-15043-7

**Published:** 2022-06-28

**Authors:** Dongli Liu, Kate Gunther, Luis A. Enriquez, Benjamin Daniels, Tracy A. O’Mara, Katrina Tang, Amanda B. Spurdle, Caroline E. Ford

**Affiliations:** 1 Gynaecological Cancer Research Group, Lowy Cancer Research Centre, School of Women’s and Children’s Health, Faculty of Medicine, University of New South Wales, Sydney, NSW 2052 Australia; 2 Medicines Policy Research Unit, Centre for Big Data Research in Health, Faculty of Medicine, University of New South Wales, Sydney, Australia; 3 QIMR Berghofer Medical Research Institute, Brisbane, Australia; 4 South Eastern Area Laboratory Services Pathology, Prince of Wales Hospital, Sydney, Australia

Correction to: *Scientific Reports* 10.1038/s41598-020-70924-z, published online 17 August 2020

The original version of this Article contained an error in Figure 4 where the α-Tubulin loading control was duplicated in panel B. The original Figure 4 and accompanying legend appear below.

**Figure 4:**
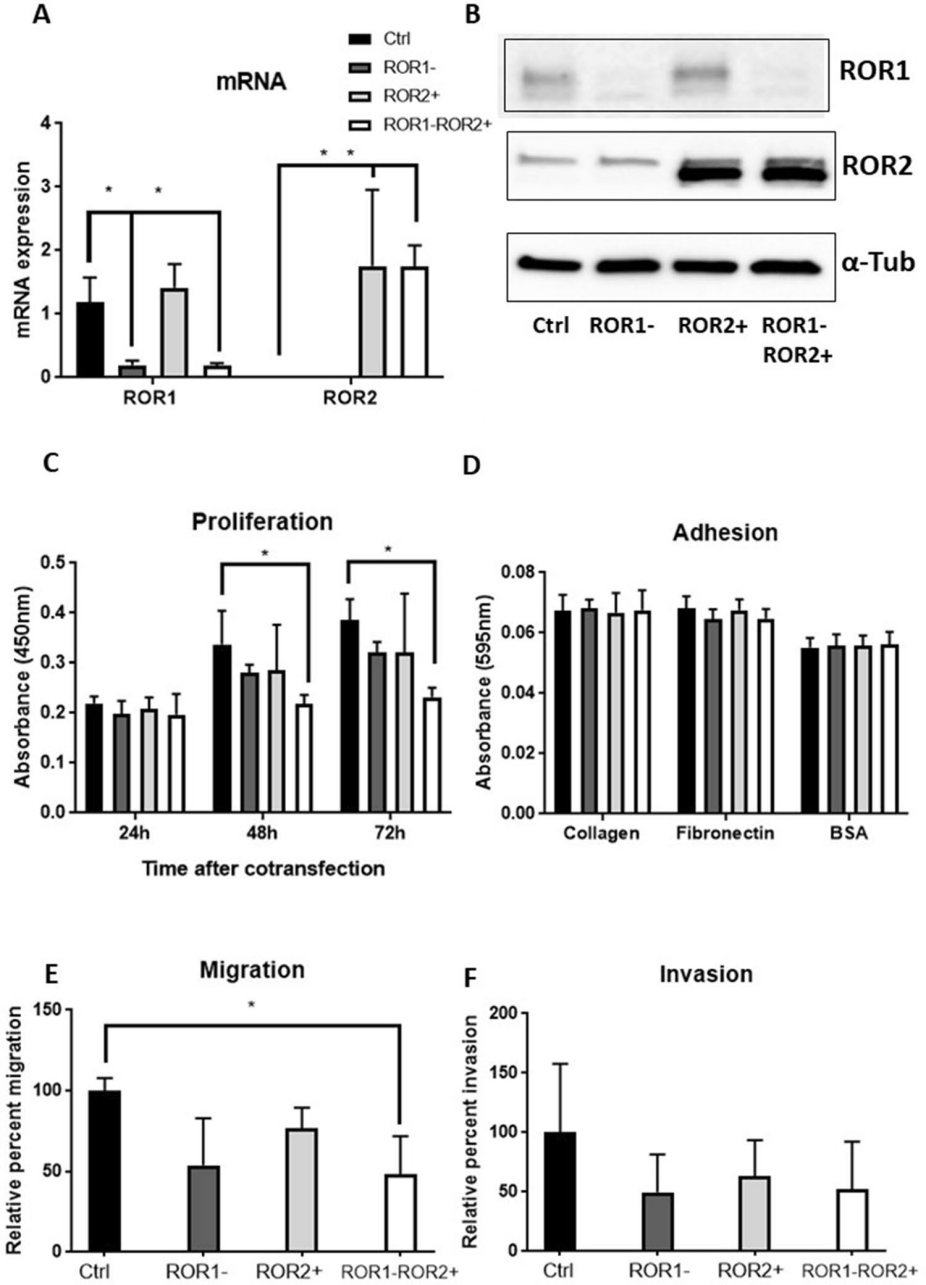
ROR1 knockdown and ROR2 overexpression significantly decreased proliferation and migration of KLE. (**A**) ROR1 mRNA expression level was reduced significantly without changing ROR2 following single ROR1 siRNA transfection. ROR2 mRNA expression level was elevated significantly with no changes in ROR1 mRNA level following single ROR2 plasmid transfection. Cotransfecting ROR1 siRNA and ROR2 plasmid significantly reduced ROR1 while increased ROR2 at mRNA level. (**B**) Representative western blot membranes showed effective delivery of ROR1 siRNA and/or ROR2 plasmid in KLE. (**C**) ROR1 knockdown and ROR2 overexpression significantly reduced the cell proliferation after 48 h and 72 h (*p* = 0.043 and 0.004 respectively). (**D**) ROR1 knockdown and/or ROR2 overexpression had no effect on adhesion to collagen or fibronectin. (**E**) ROR1 knockdown and ROR2 overexpression decreased KLE migration ability significantly (*p* = 0.037). (**F**) No significant change was observed for invasion following ROR1 knockdown and/or ROR2 overexpression. For all panels n = 3, error bars represent standard deviation of the mean, *p < 0.05.

The original Article has been corrected.

