# Supplementary material for: ROR1 is upregulated in endometrial cancer and represents a novel therapeutic target
Source: Sci Rep. 2020 Aug 17;10:13906. doi: 10.1038/s41598-020-70924-z (PMC7431863; doi:10.1038/s41598-020-70924-z)
Supplement: Supplementary file 1 — Supplementary Figures. [file 41598_2020_70924_MOESM1_ESM.docx]

**ROR1 is upregulated in endometrial cancer and represents a novel therapeutic target.**

Dongli Liu^1^, Kate Gunther^1^, Luis A Enriquez^1^, Benjamin Daniels^2^, Tracy A. O’Mara^3^, Katrina Tang^4^, Amanda B. Spurdle^3^, Caroline E Ford^1*^

^1^Gynaecological Cancer Research Group, Lowy Cancer Research Centre, School of Women's and Children's Health, Faculty of Medicine, University of New South Wales, Australia

^2^Medicines Policy Research Unit, Centre for Big Data Research in Health, Faculty of Medicine, University of New South Wales, Australia

^3^QIMR Berghofer Medical Research Institute, Australia

^4^South Eastern Area Laboratory Services Pathology, Prince of Wales Hospital, Randwick, Australia

*Corresponding author: Dr Caroline Ford, Gynaecological Cancer Research Group, Lowy Cancer Research Centre and School of Women’s and Children’s Health, Faculty of Medicine, University of New South Wales, New South Wales, 2052, Australia. T: +61293851457 F: +61293851510 E: caroline.ford@unsw.edu.au


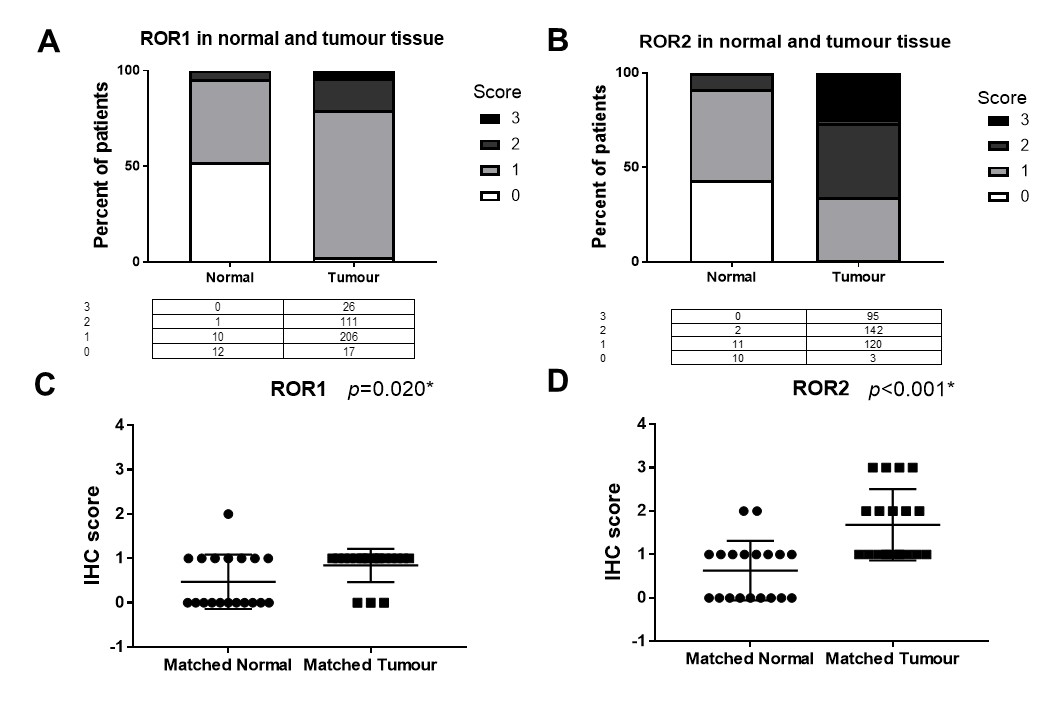


***Supplementary Fig. S1. ROR1/2 expression in normal and tumour tissue in endometrial cancer patients.*** A: Expression of ROR1 in normal (n=23) and tumour tissue (n=360). The values in the table below showed the number of score 0, 1, 2, 3 in each tissue type. B: Expression of ROR2 in normal (n=23) and tumour tissue (n=360). C: Expression of ROR1 in the matched normal and tumour tissue (n=19). *P* values resulted from paired t-test (two tailed). The expression level of ROR1 in tumour tissue is significantly different from that in the adjacent normal tissue (*p*=0.020). D. Expression of ROR2 in the matched normal and tumour tissue (n=19). The expression level of ROR2 in tumour tissue is significantly different from that in the adjacent normal tissue (*p*<0.001). *Significant at p<0.05 level.


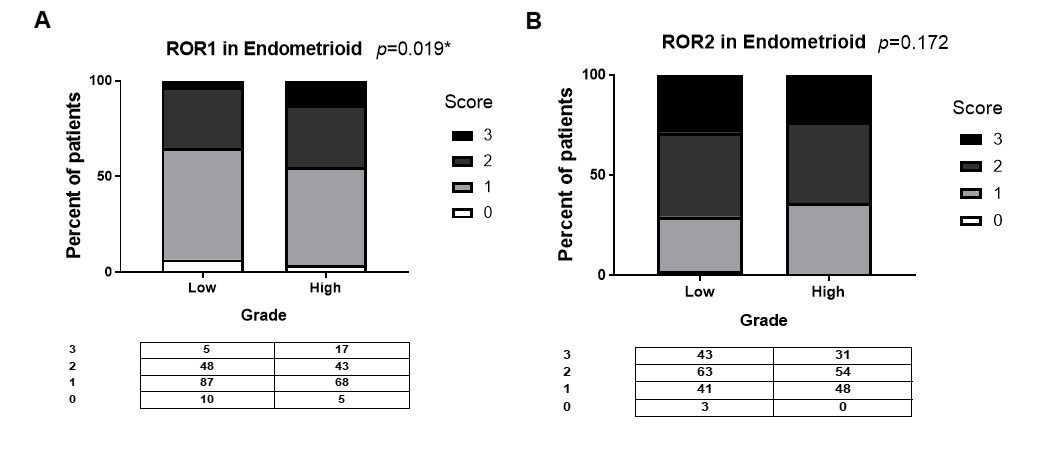


***Supplementary Fig. S2. ROR1/2 expression in endometrioid endometrial cancer patient samples stratified by grades.*** A: The expression level of ROR1 in endometrioid endometrial cancer patients was correlated with grade (*p*=0.019). The values in the table below showed the number of score 0, 1, 2, 3 in each grade. *P* values resulted from Chi-square or Fisher’s exact test indicated the significant level of the correlation. B: ROR2 expression did not differ significantly in different grades of the endometrioid endometrial cancer patients.


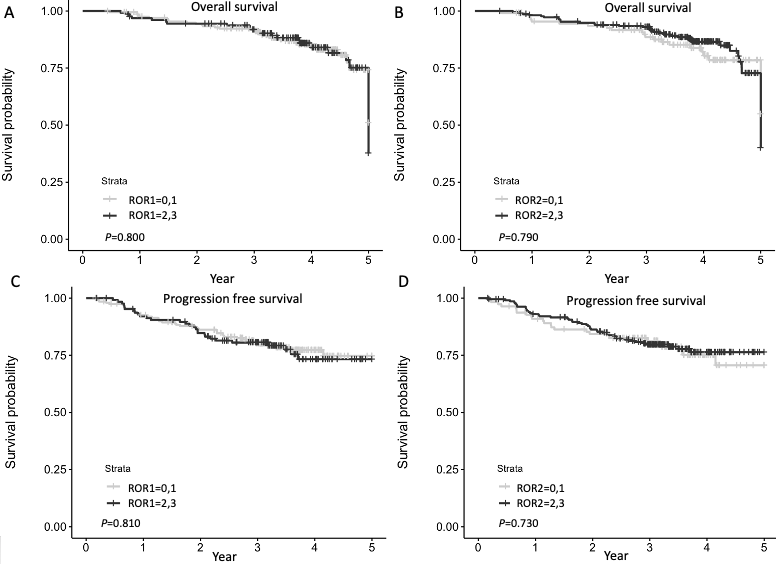


***Supplementary Fig. S3. Kaplan-Meier analysis for ROR1 and ROR2 stratified by low (score 0,1) and high (score 2,3) in the complete cohort (n=330).*** A: Overall survival (OS) according to ROR1 expression. B: Progression free survival (PFS) according to ROR1 expression. C: OS according to ROR2 expression. D: PFS according to ROR2 expression.


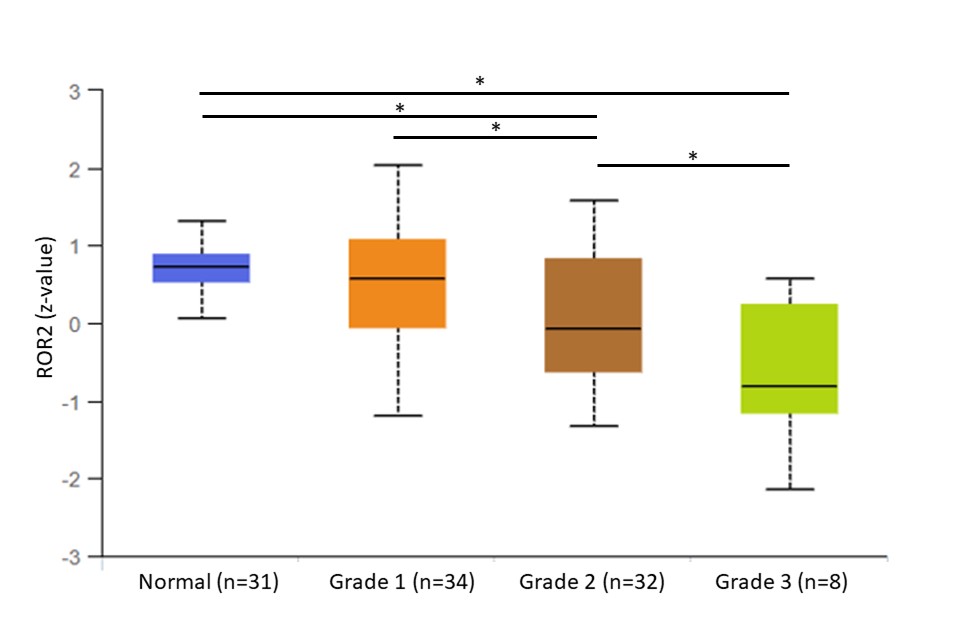


***Supplementary Fig. S4. ROR2 expression in Clinical Proteomic Tumor Analysis Consortium (CPTAC) Confirmation/Discovery cohort (n=131) stratified by endometrial cancer tumour grade adjusted from ^1^.*** Z-values represent standard deviations from the median across samples for the given cancer type. *P* values were obtained from unpaired t-test (2-tailed). *Significant at p<0.05 level.

**
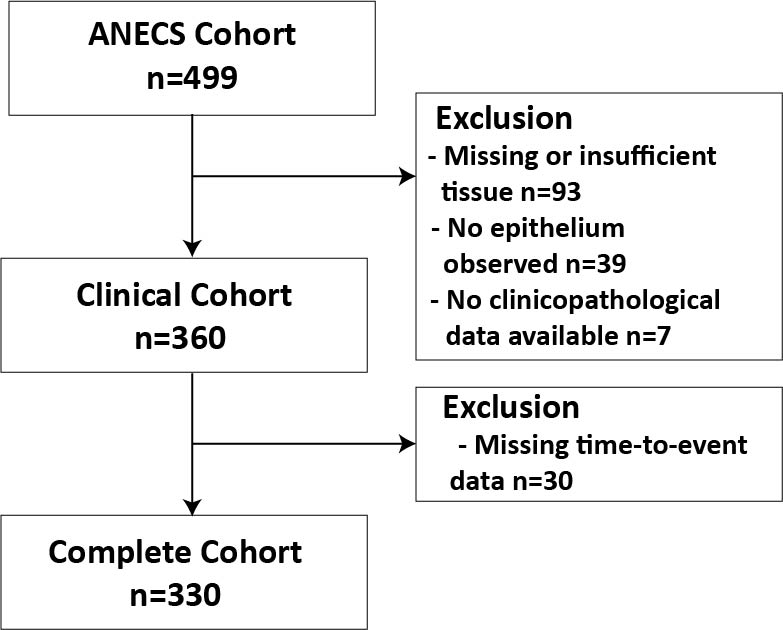
**

***Supplementary Fig. S5. Flowchart of included and excluded sample size.*** The initial ANECS cohort included samples from 499 patients. We excluded 93 samples because of missing or insufficient tissue for scoring, 39 samples for no epithelium and another 7 due to unavailable clinicopathological parameters to form the clinical cohort of 360 patients. Then we further excluded 30 people as no time to death/progression data was available for survival analysis, resulting in the complete cohort (n=330) for survival analysis.


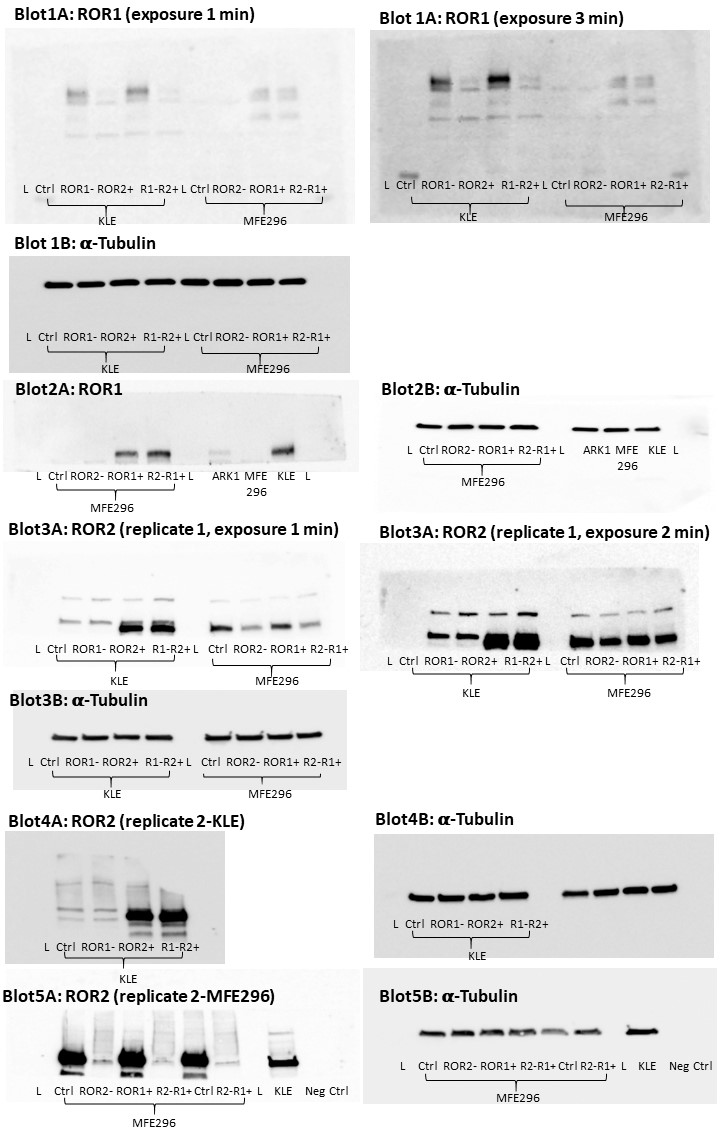


***Supplementary Fig. S6. Full-length western blots imaged by the ImageQuant LAS4000 system (GE Healthcare Life Sciences, USA).*** Blots shown in **Figure 4B** were extracted from Blot 1A, 3A and 3B. Blots in **Figure 5B** were extracted from Blot 2A, 3A and 2B. Blot 4 and 5 were replicate blots of Blot 3.

**References**

1 Chandrashekar, D. S. *et al.* UALCAN: a portal for facilitating tumor subgroup gene expression and survival analyses. *Neoplasia* **19**, 649-658 (2017).
